# Supplementary material for: Empirically validating a computational model of automatic behavior shaping
Source: PLoS One. 2025 Jan 16;20(1):e0313925. doi: 10.1371/journal.pone.0313925 (PMC11737743; doi:10.1371/journal.pone.0313925)
Supplement: Attachment — Submitted filename: Response to Reviewers.docx [file pone.0313925.s001.docx]

We thank the reviewers for their helpful comments and suggestions that have led to revisions that have improved the manuscript. Below, each reviewer’s comment is listed in **bold,** with our response immediately following it. Furthermore, to aid with the processing of these comments, all major changes implemented in the manuscript have been made with *Track Changes* activated.

**REVIEWER 1**

**1. In Figure 2 (a) Trial 1, 2, 3 and 45, it was accompanied with captions Linear, Concave Up, Concave Down, etc. However, I could not find any difference between the targets in different screen. Could you please clarify? Also, why 45 trials were chosen, is this enough? Also, was the targets were mixed with shaping function among the 45 trails, or it was continuous, like first 15 is linear, second 15 is concave up, etc.? Because the Figure 2(a) confused it up.**

Figure 2a only shows the target and reinforcement circles since showing the entire shaping functions would make the images too cluttered to understand. For this reason, the shaping functions are shown in Fig. 2b. Each participant was presented with 15 trials of each shaping function (linear, concave-up, and concave-down), resulting in 45 trials overall. The 45 trials were not blocked according to the shaping function and instead were randomly shuffled for each participant. We added the following underlined content to the figure caption to convey this information:

As each trial progressed, the reinforcement circle collapsed towards the target circle according to either a linear, concave-up or, concave-down shaping function (15 of each type, with the order of the overall 45 trials randomly shuffled for each participant). These schematic figures show only the target/reinforcement circles and do not illustrate the shaping functions, which are shown in Panel b),...

Regarding the selection of 45 trials, during pilot testing this study, we determined that interacting with each shaping schedule 15 times was sufficient to detect differences according to shaping schedule. This information is now presented in the last paragraph of *Section 2.2 Experimental Design*, via the following sentence:

Pilot data indicated that 15 exposures to each shaping function was sufficient to detect function-related differences in outcomes.

**2. Also, I do not understand why would the participants wants to click 10 times to 50 times? As I do not feel the target is too difficult to select. Just a more clarification is needed on that.**

Our primary outcome was accumulated area (AA) under the average trajectory curve for each shaping function (see Fig. 3). An unbiased calculation of this measure requires the same number of clicks in each trial, which we set to 50. However, to maintain engagement, we sought to eliminate the possibility of a participant finding the hidden target early and having to click numerous times until the trial ended (e.g. 45 clicks in the target if it was found on click 5). Therefore, once the target was encountered in 10 consecutive clicks, the trial ended and all clicks through and 50^th^ were assumed to be on target. To clarify this point, we have changed the last sentence in the last paragraph of *Section 2.2 Experimental Design*, to read:

Each trial lasted for 50 clicks, unless 10 consecutive clicks were in the target circle, which resulted in the trial being terminated early as to avoid the need for continued clicking once the target had been found.

**3. Page 15, first line 1, add period after “the AA models)”**

We have made this change and thank the reviewer for noticing this.

**4. I was initially not sure still how this experiment can translate to real-world setting specifically if that happens with people with certain disorders. But the discussion section made justice to my doubts with good examples. Since these examples came at the end of the paper, it would be ideal to have a line with multiple example with (each with one word) in the abstract, so that the readers are aware how this experiment can be helpful and continue to read till discussion.**

We have added the following sentence to the end of the abstract:

The automatic shaping routines that this study helps develop can be applied in a number of domains including exercise intensity and duration, tobacco/cannabis smoking, caloric intake, and screen time.

**REVIEWER 2**

**1. More detailed background information could be provided on how automatic behavior shaping specifically differs from traditional operant conditioning or other similar interventions. This will provide better context for readers less familiar with the field.**

We thank the reviewer for this suggestion and now better clarify that behavior shaping is a technique that an operant-conditioning framework enables. We have conveyed this by adding the following underlined content to the opening paragraph:

Operant conditioning posits that behaviors met with reinforcing consequences will be more likely to be repeated under similar contexts in the future, while behaviors met with punitive consequences will be less likely to be repeated (1). These consequences can either exist naturally (e.g., regular exercise leads to better health), or they can be engineered by an external entity (e.g., a counselor provides a monetary incentive every time their patient meets the CDC guideline of 150 minutes of moderate intensity physical activity in a week). Regarding the latter, it can often be effective to gradually guide individuals toward a goal, especially if the objective is complex or difficult to achieve. In other words, instead of waiting for a target behavior to be emitted to provide a reward, as per simple operant conditioning, it might be useful to initially establish a looser goal that eventually converges toward the final goal. Operant theory specifies this process as behavior shaping, which is the cultivation of a targeted behavior via reinforcing successive approximations to the target (2)...

**2. Highlight how this model and its validation can directly impact mHealth interventions, specifically elaborating on tangible applications.**

We agree that the paper would be improved with a more tangible example of its impact. We have therefore added the following underlined content to second paragraph of the Discussion:

The results detailed herein partially validate this computational model, making it a potentially useful tool for intervention design. For example, in the Introduction we outlined a scenario where smokers could be rewarded for abstinent time periods, with the time required for reinforcement steadily increasing. And we posed the question of how quickly the reinforcement threshold should change. The finding from the computational model, now verified by the experimental model in this study, suggests the threshold should rapidly increase at the outset of the intervention (e.g., 30-min per day over the first week) rather than more gradually. Future trials can empirically investigate this question.

**3. Consider adding a justification for using auditory tones as reinforcers instead of tangible rewards or penalties, to address concerns that this reinforcement may not fully replicate real-world scenarios.**

The use of auditory stimuli was motivated by secondhand smoke reduction interventions that we have performed which used auditory stimuli to discourage indoor smoking. We have now included this context in the first paragraph of the *Experimental Design*, via the following underlined sentence:

Participants were told to find the target area and when they clicked in the reinforcement circle, a pleasant tone was played; an unpleasant tone was played when participants clicked outside of that area. These auditory stimuli are consistent with those used in our previous mHealth behavior trials [15].)

**4. Provide further details on the interpretation of the incidence rate ratio for clicks and its practical implications for behavior shaping.**

The calculation and interpretation of incidence rate ratios (IRRs) for count (i.e., Poisson- distributed) variables like total clicks is similar to the calculation/interpretation of odds ratios for binary variables. An IRR of 2.5, means that the incidence rate (i.e. the number of events per unit time) is 2.5 times higher in the first group compared to the second. To make this clearer we added the following sentences to the second paragraph of the *Results* section (starting with “Table 2 illustrates”):

The IRR (incidence rate ratio) for the total clicks outcome represents how many more (IRR>1) or less (IRR < 1) clicks there were for one group versus another. For example, the IRR of 1.07 for ‘Linear vs. Concave-Down’ indicates that trials governed by a linear shaping function had 1.07 as many clicks as those governed by a concave-down function.

**5. Consider expanding on the rationale behind the selection of 54 participants and the specific randomization strategy used. This helps clarify whether the sample size provides adequate power for detecting differences between shaping functions.**

We have added the following text to the first paragraph of *Section 2.3 Sample Description and Experimental Procedures* that better outlines our protocol for calculating an appropriate sample size:

This sample size was based on data gathered from an independent pilot study (*n*=12) where we tested study protocols and procedures. An a priori power analysis indicated that *n*=48 participants were sufficient to power our study to 0.9 with a significance level of 0.05. This was performed by considering participants’ average number of clicks for each shaping function, fitting a within-factors, repeated measures ANOVA model to calculate the effect size (Cohen’s *f*=0.33), and using G*Power for a power analysis associated with this model and effect size. We opted for *n*=54 participants, which allowed for a loss of data of up to 10% of participants while still adhering to our power analysis requirements. Note that data from pilot participants was not included in any of the analyses presented in this paper.

We also note that sample size considerations are typically used to contextualize non-significant findings, and the significant findings presented in this paper represent evidence that the study was appropriately powered.

Lastly, regarding randomizing participants, this study was a within-subjects design, where every participant was exposed to every condition (i.e. shaping function). Therefore, randomization was not necessary. We now note this in *Section 2.2 Experimental Design*, where the following underlined text was added.

The trial was a within-subjects design (all participants experience each condition) where each participant completed 45 target location trials, which were randomly assigned to each of the three shaping functions, such that participants were exposed to each function 15 times

**6. Elaborate on why three specific shaping functions (concave-up, linear, concave-down) were selected and how they represent a comprehensive spectrum of reinforcement criteria.**

The three selected shaping functions are representative of three qualitative classes from the computational experiment, namely: consistent narrowing (linear), rapid narrowing (concave up), and gradual narrowing (concave down) of the reinforcement window. The outcomes observed in the computational experiment were on a continuum and did not have strong nonlinear effects, which indicates that the findings associated with these representative functions are likely to be robust. But further work is required to tease apart subtleties associated with different reinforcement functions. This point has now been made clearer in the manuscript via the following sentence added to *Section 2.2 Experimental Design:*

These functions represent three distinct qualitative shaping classes studied in the computational experiment: consistent narrowing of the reinforcement window (linear), rapid initial narrowing (concave up), and gradual initial narrowing (concave down). The use of three functions was appropriate to examine the broad-scale correspondence between computational and experimental outcomes, while more nuanced investigations (e.g. the impact of slightly vs. extremely concave down functions) are left for future work.

**7. Clarify specific recommendations for practitioners or researchers looking to implement automatic behavior shaping in different settings, including potential limitations or challenges.**

We thank the reviewer for this suggestion. The fourth paragraph of *Section 4 Discussion* included some discussion of limitations associated with automatic behavior shaping. We have now added the following underlined text to convey additional challenges:

While this is an unprecedented opportunity to increase the precision and reach of intervention science, there are several challenges associated with implementing automatic shaping routines, chief among these being the ability to objectively measure behavior and provide continual feedback. Such procedures require ample planning to navigate technological and operational hurdles, as well as forethought concerning the potential intrusiveness of ongoing participant monitoring. A related obstacle is the potential for participants to negatively react to the long-term observation of their behavior, which might be viewed as surveillance. Consequently, it is critical that automatic shaping trials follow recommendations for e-health trials to include human support that is perceived as being trustworthy, benevolent, and having expertise (18). Furthermore, we recognize that the automatic systems will not be able to replicate the nimble and complex processes that teachers, coaches, counselors, and other professionals regularly execute to shape behavior. However, if automatic shaping systems can capture a rudimentary approximation of human-implemented procedures and achieve some level of their success, the scalability offered by mHealth tools (19) has the potential to enable significant gains in public health.
