# Supplementary material for: Empirically validating a computational model of automatic behavior shaping
Source: PLoS One. 2025 Jan 16;20(1):e0313925. doi: 10.1371/journal.pone.0313925 (PMC11737743; doi:10.1371/journal.pone.0313925)
Supplement: Attachment — Submitted filename: Response to Reviewers.docx [file pone.0313925.s002.docx]

We thank the reviewers for their helpful comments and suggestions that have led to revisions that have improved the manuscript. Below, each reviewer’s comment is listed in **bold,** with our response immediately following it. Furthermore, to aid with the processing of these comments, all major changes implemented in the manuscript have been made with *Track Changes* activated.

**REVIEWER 2**

**Highlight the practical implications of the findings for health behavior interventions, emphasizing how the concave-up shaping functions could be utilized in real-world applications.**

Generally speaking, at the outset of shaping a behavior, one can proceed by either i.) slowly narrowing the reinforcement criteria towards the target goal, which theoretically provides participants with a history of reinforcement that allows them to identify the precursors of this outcome or ii.) rapidly narrowing the reinforcement window so that the target is encountered and reinforced as quickly as possible, which risks participants struggling to perform the target behavior and receive the associated benefits. Our finding that concave-up shaping functions perform best, both in computational and laboratory experiments, suggests that the latter is a better strategy, which is important information to have when designing health-behavior interventions. The following underlined content was added to the second paragraph of *Section 4 Discussion* to better clarify this point:

The finding from the computational model that concave-up shaping functions best induce shaping, now verified by the experimental model in this study, suggests the threshold should rapidly increase at the outset of the intervention (e.g., 30-min per day over the first week) rather than more gradually. This further suggests that shaping in other contexts (e.g. requiring progressively more daily physical activity to receive reinforcement) would also benefit from aggressively tightening reinforcement criteria at the beginning of the study, despite the risk that participants may struggle to perform the target behavior. Future trials should empirically investigate this question within a health intervention setting.

**2. Expand on the background information to provide a more comprehensive understanding of the significance of automatic behavior shaping in health interventions. Include recent advancements and challenges in the field.**

**8. Discuss the results in the context of existing literature. Highlight how the findings support or challenge previous studies and the implications for designing automated behavior-shaping interventions.**

These two comments represent requests to tie the study’s aims and results to existing literature regarding automated behavior-shaping interventions, particularly as they relate to previous advances and challenges in this field. We note that we are the forefront of highlighting the unique opportunity that wearables and environmental sensors enable for automatic behavior shaping within the field of health behavior interventions. Consequently, there are few examples in the literature that discuss automated behavior shaping, which we are aiming to change via the publication of this manuscript. While some previous research has identified behavior shaping as a theoretical approach with promise in a clinical setting,^[1]^ shaping (and other operant approaches) have largely been discussed within the field of applied behavior analysis (ABA) for addressing developmental disorders (e.g. Ref.^[2]^). There have been occasional calls to integrate shaping into applied settings^[3]^, but the larger health intervention field has generally focused more on antecedent approaches (e.g. knowledge building, behavior prompts) than post-cedent strategies like behavior shaping. As mentioned in the manuscript, advances in technology have allowed behavioral contingencies to be automated, which opens the door for formal behavior shaping to be automated. A major goal of this paper is to demonstrate that automated behavior shaping can be engineered in human subjects to generate targeted behavior responses, which we had already shown in a computational context. Our intention is that through work like ours, behavioral interventionists will begin to recognize the potential of behavior shaping as an intervention strategy and begin to incorporate it into trials.

To better clarify these points, we have added the following underlined content to the paper, which includes more citations that provide background information. First, in the second paragraph of *Section 1 Introduction*, we now state:

In general, health interventions have historically focused on antecedents of behavior (e.g., knowledge, prompts) rather than post-cedent features that enable operant phenomena like behavior shaping. (5) While behavior shaping has largely been implemented via applied behavior analysis to address development disorders (6), there have been calls for its integration it into more general settings. (7,8)

In the third paragraph of the Introduction, we also state:

Because automatic shaping is a nascent field, its potential alignment with mHealth is not widely recognized and the specifics of how to best arrange shaping procedures are unknown ... To demonstrate the potential effectiveness of automatic behavior shaping and to preliminarily investigate how to best arrange autonomous shaping procedures, Berardi et al. performed computer simulations...

**3. Clearly articulate the gap in current research that this study aims to fill. Specify why validating the computational model with human subjects is critical for advancing the application of behavior-shaping technologies.**

**4. State the research objectives explicitly, highlighting the importance of translating computational models into experimental settings.**

As suggested by these two comments, to more clearly convey our aims for the study and outline the benefits of achieving these objectives, the following underlined content was added to the last paragraph of *Section 1 Introduction*:

…Therefore, the degree to which the predictions made by the computational shaping model generalize to human behavior is unknown. Establishing this link is a critically important step towards behavior shaping becoming a widely deployed technique in mHealth promotion interventions. To address this knowledge gap, we developed and performed a laboratory behavioral experiment that mimicked key features of the computational behavior shaping model. Our aim in performing this work is two-fold. First, we seek to highlight the general principles underlying behavior shaping and to outline considerations for its implementation (e.g. the continued updating of reinforcement criteria). Second, by establishing a correspondence between laboratory and digital experiments, we will demonstrate that the latter approximates real-world behavior shaping outcomes to a sufficient degree to make it a suitable tool for efficiently sandboxing behavior-shaping procedures before deploying them within health promotion trials.

**5. Provide more detailed information about the participants, such as demographic characteristics and any relevant inclusion or exclusion criteria. This will help readers understand the sample's representativeness.**

As described in the *Sample Description and Experimental Procedures*, participants were undergraduate students at a university who were required to be enrolled in in the university’s student research pool, typically for the purpose of receiving credit for a research requirement in an introductory Psychology class or as extra credit. They were also required to process the software instructions in English and have no visual or hearing impairments. We now better clarify the demographic and inclusion/exclusion criteria in *Section 2.3* *Sample Description and Experimental Procedures* as follows:

Participants were recruited between May 3, 2019, and November 11, 2019, using the SONA Experiment Management System (SONA), a university platform designed to recruit students from courses with research participation requirements as part of their curriculum. Eligibility criteria included enrollment in the university’s SONA subject pool, the ability to understand English software instructions, and the absence of any visual or hearing impairments. Upon completing the study, participants received credit in line with their course policies. Of the 54 students in the study, 30 were female and 24 were male.

**6. Elaborate on the experimental setup, including how the target and reinforcement circles were programmed and the rationale behind choosing specific shaping functions. Consider including a diagram or flowchart to illustrate the experimental procedure.**

We agree that a flowchart summarizing the experimental procedures would be useful and have added this to Figure 2. After making this change, we decided to split Figure 2a and Figure 2b into Figures 2 and 3, with the former focusing on experimental procedures and the latter illustrating shaping functions.

Additionally, we reframed the information regarding the programming of the target and reinforcement criteria by adding the following underlined text to the last paragraph of *Section 2.2: Experimental Design*:

At the beginning of each trial, the shared center of the target and reinforcement circles was randomly determined using Matlab's random number generator, with the constraint that the entire reinforcement circle remained within the screen. Pilot testing indicated that a 100 pixels (px) radius for the target circle and 400px radius for the reinforcement circle were appropriate. In contrast with the computational experiments, the starting width of the reinforced area was not systematically varied (i.e. it was fixed at 400px in every trial), since resources were limited and computational outcomes were not sensitive to this parameter. Each trial consisted of 50 clicks, but to prevent redundant clicking after the target was located, trials ended early if 10 consecutive clicks landed within the target circle.

The rationale behind the selection of shaping functions is provided in the second paragraph of *Section 2.2: Experimental Design*, which has been modified as follows to improve clarity:

While the computational simulations tested 41 different values of *b*, replicating this in a real-world experiment would have been too resource intensive. Instead, we focused on three specific cases: i.) *b* = -0.3, representing a concave-up function; ii.) *b* = 0.3, representing a concave-down function; and iii.) *b* ≈ 0, which, after a linear Taylor expansion, corresponds to the function *r(n)* = -30*n* + 400. These cases capture three distinct patterns explored in the simulations, namely: i.) rapid initial narrowing (concave up), which leads to the target being encountered and reinforced as quickly as possible, but risks participants struggling to perform the target behavior and receive the associated reward; ii.) slower initial narrowing (concave down), which provides participants with a steady history of reinforcement that allows them to identify the antecedents of this outcome; and iii.) steady linear narrowing of the reinforcement window, which is a compromise between the first two phenomena. This approach allowed us to broadly compare computational and experimental results, while leaving more detailed investigations (e.g., slight versus extreme concave-down functions) for future research. See Fig. 3 for an illustration of the shaping functions.

**7. Provide a more detailed explanation of the statistical methods used, including any assumptions made and how they were tested. Consider including additional analyses, such as effect size calculations, to enhance the interpretation of the findings.**

We thank the reviewer for this suggestion. We have now added details concerning our assessment of the assumption that residuals are approximately normally distributed and our selection of a negative binomial versus Poisson regression model. Furthermore, when revisiting these models, we determined that the mixed effects model for total clicks as the dependent variable was unnecessarily complex, as the variance accounted for by random effects was negligible. Consequently, we decided to remove the random effects and instead fit a generalized linear regression model. The new modeling results were similar to those from the previous model (see updates in Table 2). However, this simpler approach will be easier for readers to grasp. Lastly, in terms of effect size, we provide the percent of variance explained by the shaping functions for the mixed effects model and the incidence rate ratio for the generalized linear regression model. These changes were made through the addition of the underlined content added to *Section 2.5 Statistical Analysis* as follow:

... More specifically, using a random-intercept, Gaussian mixed-effects model, the shaping function was regressed on AA, with participant ID serving as the random effect. The variance partition coefficient was calculated, which indicates the proportion of total variance in the dependent variable that is accounted for by the shaping functions, which serves as a measure of effect size.

When regressing the total number of clicks on the shaping function , we initially fit a generalized mixed effects model as above (but with a negative binomial outcome due to the count nature of the dependent variable), with participant ID and trial number serving as completely crossed random effects. However, the proportion of variance explained by the random effects was < 0.01, so we elected to proceed with a more parsimonious generalized linear regression model without a hierarchical structure. While count variables can be modeled via Poisson regression, this approach assumes that the mean and standard deviation of the dependent variable are equal. The dispersion parameter can be used to assess this assumption, which for our model was 8.33. This value is much larger than the dispersion parameter near 1 that is expected for Poisson models and indicates overdispersion (variance larger than mean), making the negative binomial model appropriate. The effect size for this model is given by the IRR (incidence rate ratio), which represents how many more (IRR>1) or less (IRR < 1) clicks there were for one group versus another.

For both models, the normality of residuals was confirmed visually via Q-Q plots and density estimates of the residuals. The linear shaping function was set as the referent level in our models, and a contrast matrix was used to examine the difference between results for concave-up and concave-down shaping.

We also made the following changes in *Section 3*, better clarifying the effect sizes in our results*. Results*:

The fitted variance components in the mixed effect regression models for the AA outcome allows conclusions to be drawn about the source of variance within observations and serves as a measure of effect size. A 0.63 proportion of the total variance was due to between subject differences, while 0.37 was due to the shaping functions

1. Elder, J. P., Ayala, G. X., & Harris, S. (1999). Theories and intervention approaches to health-behavior change in primary care. *American journal of preventive medicine*, *17*(4), 275-284.
2. Cihon, J. H. (2022). Shaping: A brief history, research overview, and recommendations. *Handbook of Applied Behavior Analysis Interventions for Autism: Integrating Research into Practice*, 403-415.
3. Galbicka, G. (1994). Shaping in the 21st century: Moving percentile schedules into applied settings. *Journal of applied behavior analysis*, *27*(4), 739-760.
